# Proposing Music-based Interventions for the Treatment of Traumatic Brain Injury Symptoms: Current Evidence and Future Directions

**DOI:** 10.1177/07067437211007811

**Published:** 2021-04-08

**Authors:** Adriano Mollica, Michael Thaut, Matthew J. Burke

**Affiliations:** 1 Harquail Centre for Neuromodulation and Hurvitz Brain Sciences Program, Sunnybrook Research Institute, Toronto, Ontario, Canada; 2 Neuropsychiatry Program, Department of Psychiatry, University of Toronto, 71545 Sunnybrook Health Sciences Centre, Ontario, Canada; 3 Music and Health Research Collaboratory, Faculty of Music, 7938 University of Toronto, Ontario, Canada

**Keywords:** music therapy, music-based interventions, traumatic brain injury, concussion, cognitive rehabilitation

Regardless of the classification of initial injury severity, traumatic brain injury (TBI) can result in debilitating neurologic and psychiatric symptoms that may last months to years.^1^ These post-TBI symptoms can vary widely from patient to patient, but core symptoms involve depressed mood and cognitive impairment.^1^ The underlying pathophysiology of persistent cognitive dysfunction following TBI has yet to be fully understood, but disruptions in large scale neural networks, particularly those governing resting state functional connectivity (e.g., default mode network) and cognitive control (e.g., salience network), are strongly implicated across TBI severities.^2^ Furthermore, the presence of post-traumatic depression may have bi-directional interactions on prolonging the overall recovery process.^3^ Given the deficits in multiple domains of functioning following TBI, novel rehabilitation approaches that can target multiple symptoms simultaneously are needed for this complex neuropsychiatric patient population.

Music-based interventions (MBIs) are emerging as a new potential treatment strategy for neurologic^4^ and psychiatric^5^ patient populations, as they are safe, economic, and can be creatively tailored to meet specific functional goals. MBIs are typically selected and delivered by a credentialed music therapist based on empirically supported models and can involve active (improvisation, singing, clapping, or dancing) and/or receptive (purposeful music listening to identify emotional content emerging from music) techniques.^4,5^ Mechanistically, MBIs appear to engage both cortical and subcortical areas governing attention, working memory, planning, and flexibility and can modulate these areas over time.^4^

## MBIs for TBI

A systematic review and meta-analysis published in 2020 by Mishra et al.^6^ identified 6 studies of patients with moderate–severe TBI that compared the effect of MBIs for rehabilitation to controls. Five studies were of low quality and 1 study was very low quality. Outcomes were focused on recovery of motor symptoms and on cognitive functioning. Three studies reported significant improvements in gait velocity and stride length with small overall effect. Mixed results were reported for cognitive outcomes with no significant improvement in memory (one study reporting overall worsening of memory), and 3 studies reporting improvement in executive functioning with small overall effect. Finally, only 2 of the included studies evaluated changes in depressed mood following MBIs, and although both reported statistically significant improvements, the heterogeneity of outcome measures used between these studies limits any generalizability. Overall, this meta-analysis was limited by the low number of studies, low study quality, small sample sizes (*n* < 30) within included studies, heterogeneity in outcome measures used, and lack of follow-up data.^6^

A recent crossover randomized control trial not included in the above systematic review found significant improvements in general executive functioning and set shifting skills in moderate-severe TBI (*n* = 39) following 3 months of MBIs.^7^ Each session occurred twice weekly and included three 20-minute modules involving rhythmic training (playing sequences of rhythms on a drum), structured cognitive-motor training (playing musical exercises on drum set with different movement in composition elements while accompanied by the MT on piano), and assisted music playing (learning to play participants’ favorite songs on piano). The executive function improvements were maintained at 6-month follow up, and the investigators found significant increases in grey matter volume in the right inferior frontal gyrus, which correlated with improvements in set shifting.^7^ This evidence further supports that MBIs can influence cognitive outcomes following TBI and that this could be due to engagement of corresponding neural networks.

Although injury severity was not reported in the following study, Gardiner and Horwitz^8^ reported significant improvements in planning (as measured by a series of mazes from the Weschler Intelligence Scale-III), and mental flexibility (Trail making test-B) in 22 veterans with TBI after employing specific MBI protocols targeting attention, executive function, and memory. Notably, the conclusions of these findings are limited by the open-label pretest–posttest design with no randomization or control/comparator group.

Only one study was found that evaluated the effects of MBIs on mild TBI. Vik et al.^9^ evaluated the effects of biweekly 30-minute piano instruction for patients (*n* = 7) following mTBI compared with 2 healthy control groups (musicians, *n* = 11, and non-musicians, *n* = 12). Piano exercises gradually progressed in difficulty, and patients were additionally required to practice at home for 15 minutes per day. All 7 mTBI patients had received traditional cognitive rehabilitation during their hospital stay without improvement and were all on leave from work, despite being on average 2 years post injury. Patients with mTBI not only experienced significant improvements in California Verbal Learning Test performance, but 6 of the 7 patients returned to work in their full pre-morbid capacity. Furthermore, these clinical changes were coupled with increased connectivity between right middle prefrontal cortex, right anterior insular cortex, left rostral anterior cingulate cortex, and the right supplementary motor cortex,^9^ which are important nodes in the salience and frontal-executive neural networks.^2^

## Conclusions and Future Directions

Cognitive impairment and depression in TBI are commonly reported symptoms and there are limited interventions available to effectively manage them.^1,3^ MBIs are emerging as a novel multimodal therapeutic strategy with the potential to target several symptom domains simultaneously.^4^ There is promising evidence to suggest that MBIs may have potential in rehabilitating cognitive impairments across various levels of TBI severity with most evidence for moderate–severe TBI.^6,7^ However, conclusions on efficacy are limited at this time given the lack of randomized trials for each level of injury severity (e.g., only 1 study for mild TBI^9^) small sample sizes, lack of active control groups, and overall poor study quality. It is possible that MBIs exert their effects by engaging dysfunctional neural networks implicated in TBI, but at this time, only 2 studies have investigated this relationship with fMRI; one for mild TBI^9^ and one for moderate–severe.^7^

With regards to post-TBI depression, only 2 studies included in the meta-analysis for moderate–severe TBI included mood outcomes, but they both reported statistically significant improvements.^6^ MBIs have been shown to have a large effect with moderate quality evidence for depression (not specific to TBI) according to a recent Cochrane review.^5^ Since depression is recognized as a common symptom across TBI severity that impacts functional outcomes,^1,3^ it is critical that future studies of MBIs for TBI include pre–post measures of validated depression scales.

Overall, further studies are needed to determine if MBIs can demonstrate efficacy with randomized controlled trial designs and to further understand underlying neurobiological mechanisms of this therapy with use of pre/post neurophysiological measures. Preferably, future studies will employ traditional cognitive rehabilitation strategies as a comparator group, as the above studies only compared MBIs to standard care or waitlist, as well as longer follow-up periods and evaluation of transfer to real-world functioning. Given that TBI-related cognitive impairment has limited treatment options and MBIs have no major risk of harm, we would recommend that integrated inpatient and outpatient treatment programs for moderate-severe TBI consider incorporating MBIs into their clinical management plans. The relative lack of available evidence of MBIs for mild TBI limits the generalizability of recommendations at this time, although, given the safety profile and similar limited treatment options, MBIs could be offered where resources are available. Ideally, delivery of MBIs would be carried out by a trained music therapist in collaboration with either an occupational or physical therapist following a validated protocol.^8,9,7^ There is potential to deliver MBIs by a credentialed music therapist virtually as well. However, many communities may not have access to therapists of specialized resources, and modified protocols could be developed that are self-directed (e.g., learning an instrument via internet or phone-based applications, memorizing lyrics and singing along to favourite songs, or tapping along to the beat while listening to their favourite pieces of music, for at least 15 minutes daily), although collaboration among local university-based music therapy departments (if applicable) would be recommended. Additional resources and information on university-based music therapy programs and credentialed therapists can be found at https://www.musictherapy.ca/ or www.nmtacademy.co.
